# Supplementary material for: Transcriptomics reveal stretched human pluripotent stem cell-derived cardiomyocytes as an advantageous hypertrophy model
Source: J Mol Cell Cardiol Plus. 2022 Nov 12;2:100020. doi: 10.1016/j.jmccpl.2022.100020 (PMC11708431; doi:10.1016/j.jmccpl.2022.100020)
Supplement: Supplementary file 3 — Supplementary material [file mmc3.pdf]

Supplementary Information

Transcriptomics reveal stretched human pluripotent stem cell-derived cardiomyocytes as an advantageous hypertrophy model

Lotta Pohjolainen[1], Heikki Ruskoaho[1], Virpi Talman[1]

[1] Drug Research Program and Division of Pharmacology and Pharmacotherapy, Faculty of Pharmacy, University of Helsinki, FI-00014 Helsinki, Finland

Correspondence: Virpi Talman, Ph.D., Division of Pharmacology and Pharmacotherapy, Faculty of Pharmacy, University of Helsinki, PO Box 56, FI-00014 Helsinki, Finland. E-mail: virpi.talman@helsinki.fi. ORCID: 0000-0002-2702-6505

Contents:

Supplementary Figures 1–7, p. 2–7

Supplementary Tables 1–5, p. 8–12

Supplementary Dataset Legends, p. 13

References, p. 13–14

Supplementary Figures

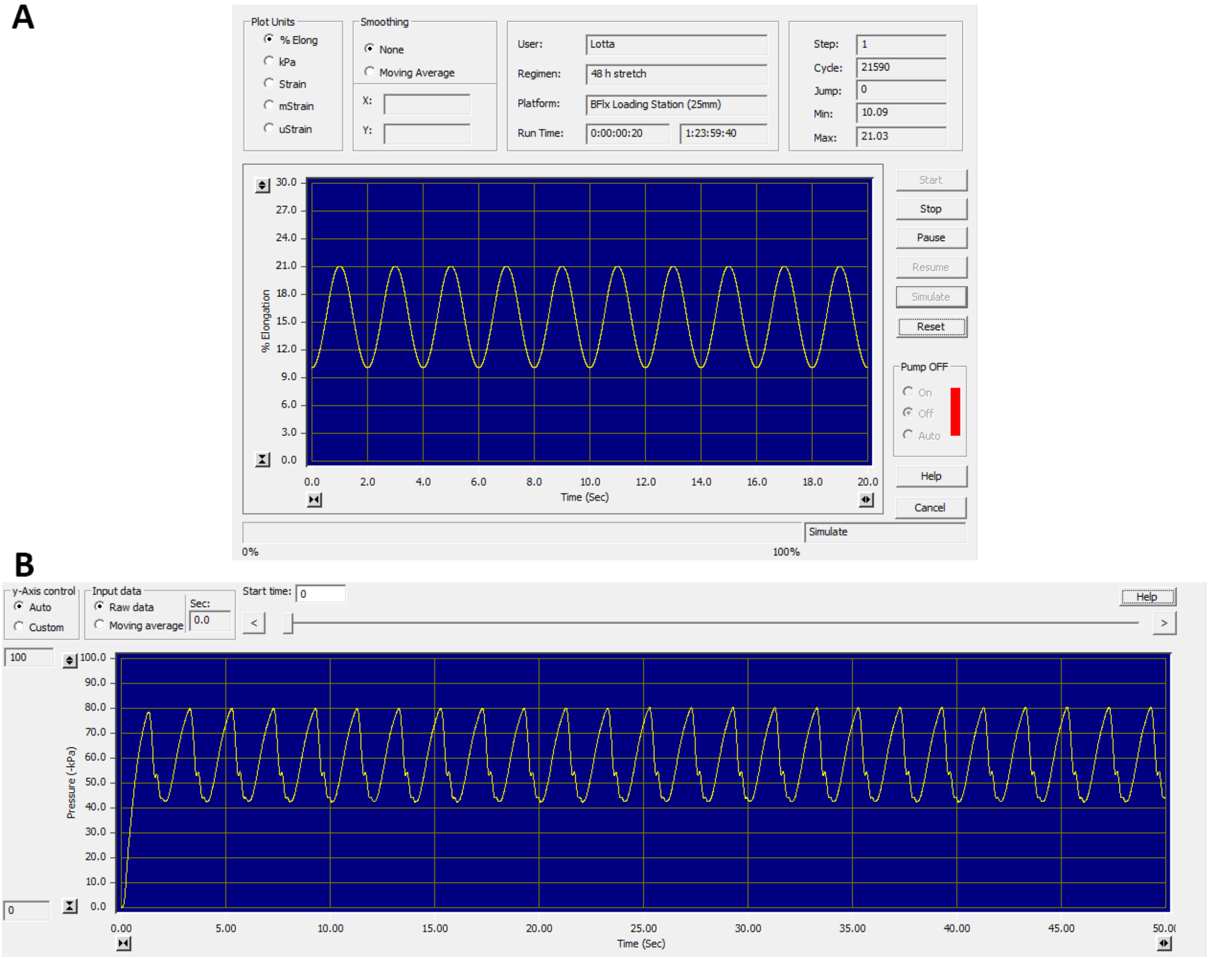

Supplementary Fig. 1. A representative graph of waveform and degree of stretch. A, A graph representing the start of a simulated regimen, where the membrane elongation (%) is plotted over time (sec). **B**, A graph representing the start of an actual regimen run, where the pressure (-kPa) is plotted over time (sec). Graphs are exported from the FX-5000™ software.

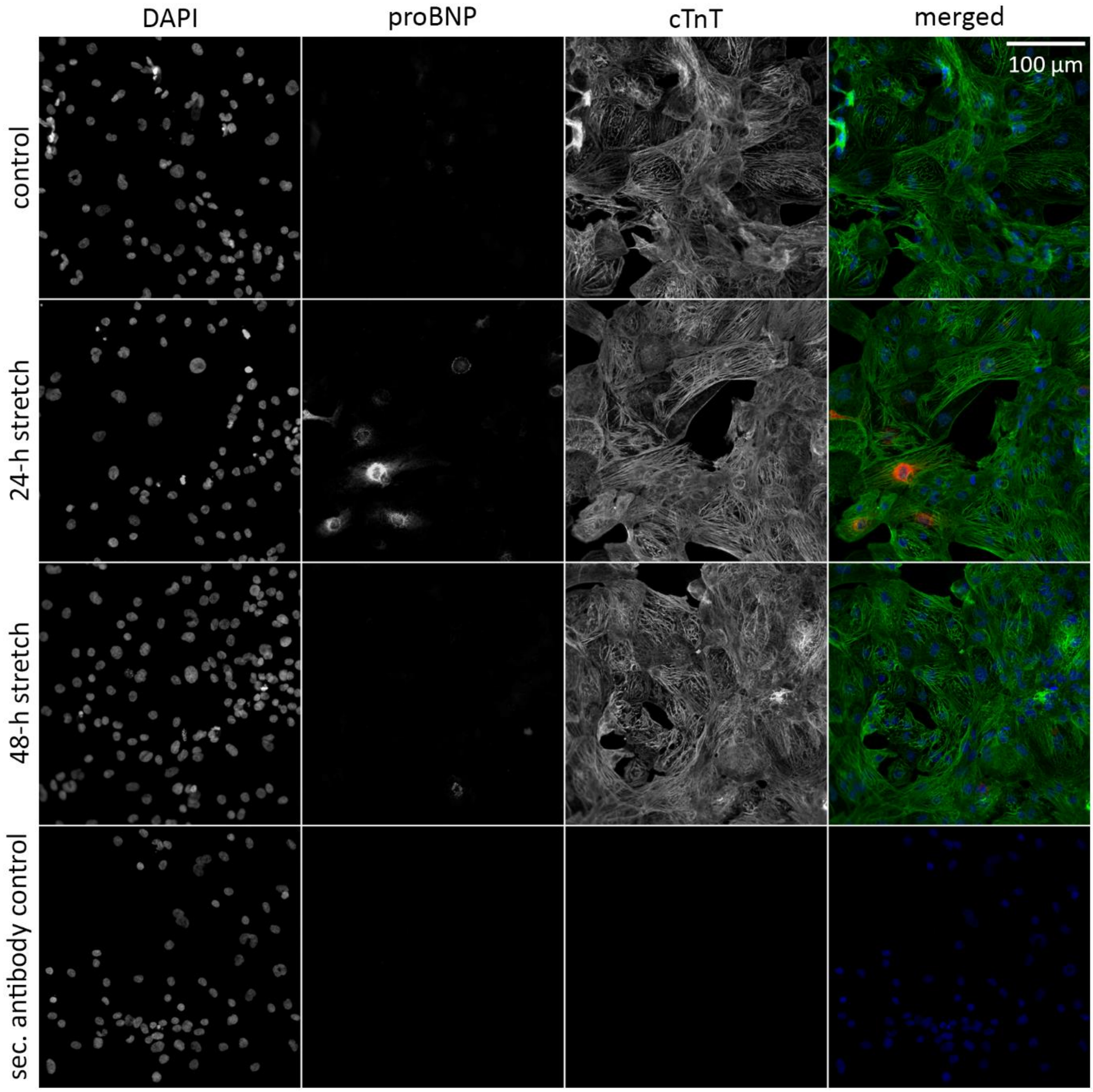

Supplementary Fig. 2. Representative images of unstretched control, 24-h stretched, and 48-h stretched hiPSC-CMs. Images are acquired with 40x water immersion objective. hiPSC-CMs were stained for DNA (DAPI; blue), pro-B-type natriuretic peptide (proBNP; red) and cardiac troponin T (cTnT; (green). Scale bar, 100 µm.

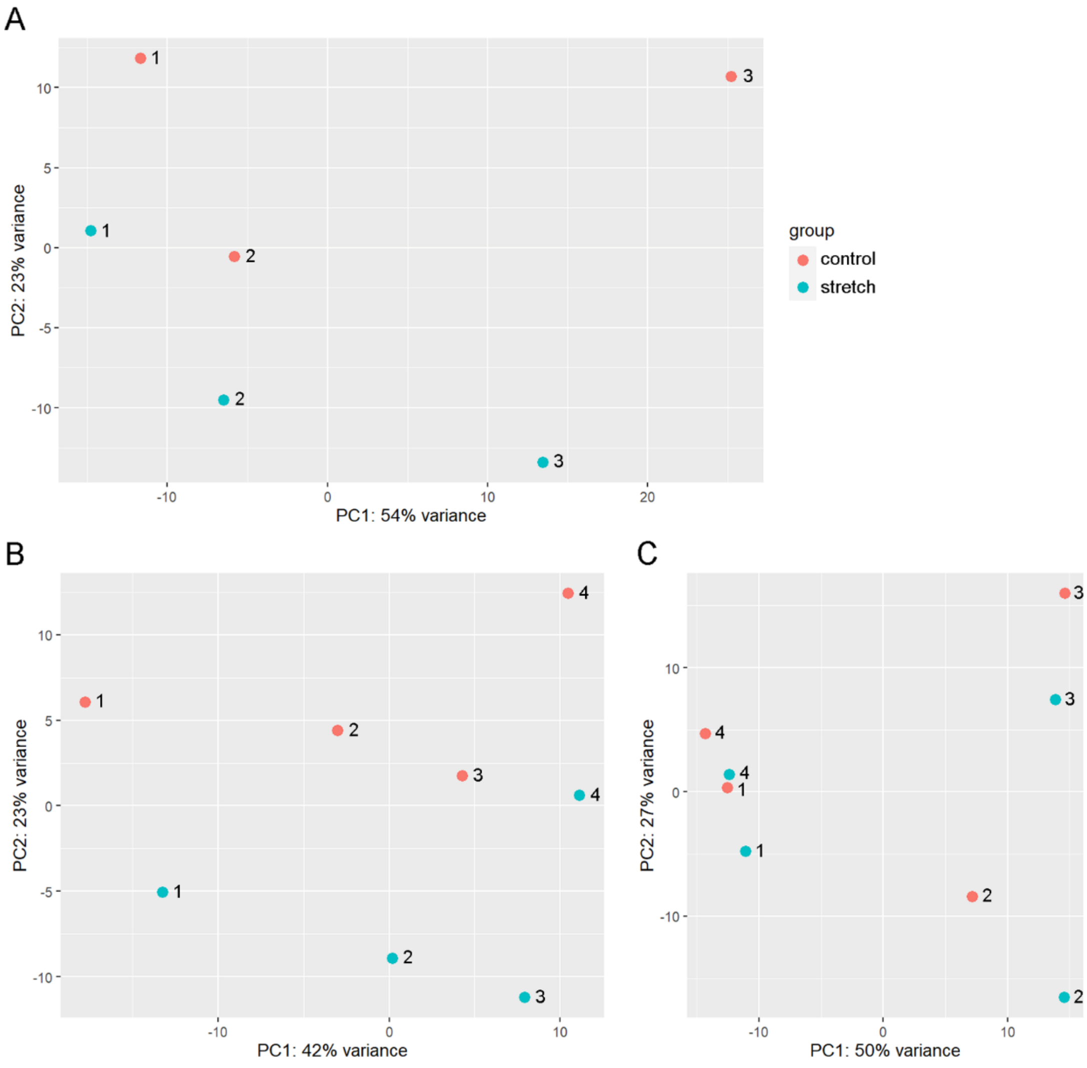

Supplementary Fig. 3. Principal component (PC) analysis plots of the RNAseq results of the stretched and the unstretched control hiPSC-CMs at 24 h (A), 48 h (B) and 72 h (C). Each dot indicates a sample, and each sample pair is numbered (1–4). n=3 for 24h, n=4 for 48h and 72h.

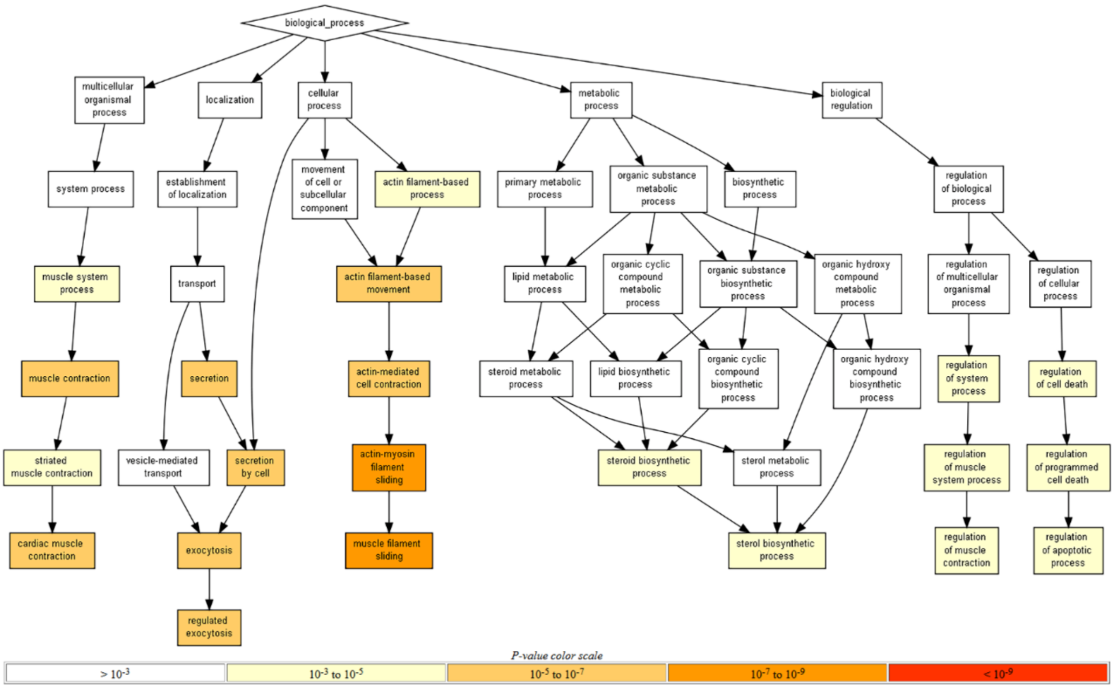

Supplementary Fig. 4. Biological processes enriched in upregulated genes after 24 h, 48 h or 72 h of cyclic stretch in hiPSC-CMs. Gene Ontology (GO) enrichment analysis was performed with GOrilla. Directed graph of enriched processes is color-coded based on the significance of enrichment.

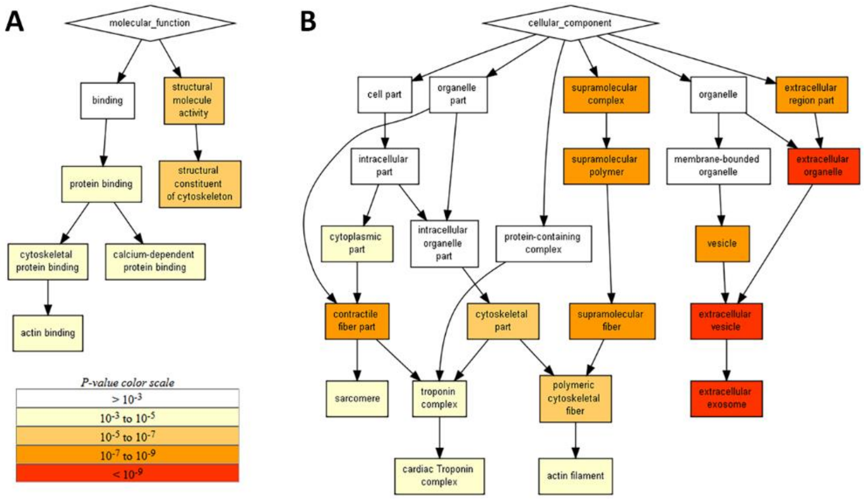

Supplementary Fig. 5. Molecular functions (A) and cellular components (B) enriched in upregulated genes after 24-h, 48-h or 72-h cyclic stretch. Gene Ontology enrichment analysis was performed with GOrilla. Directed graphs of enriched molecular functions and cellular components are color-coded based on the significance of enrichment.

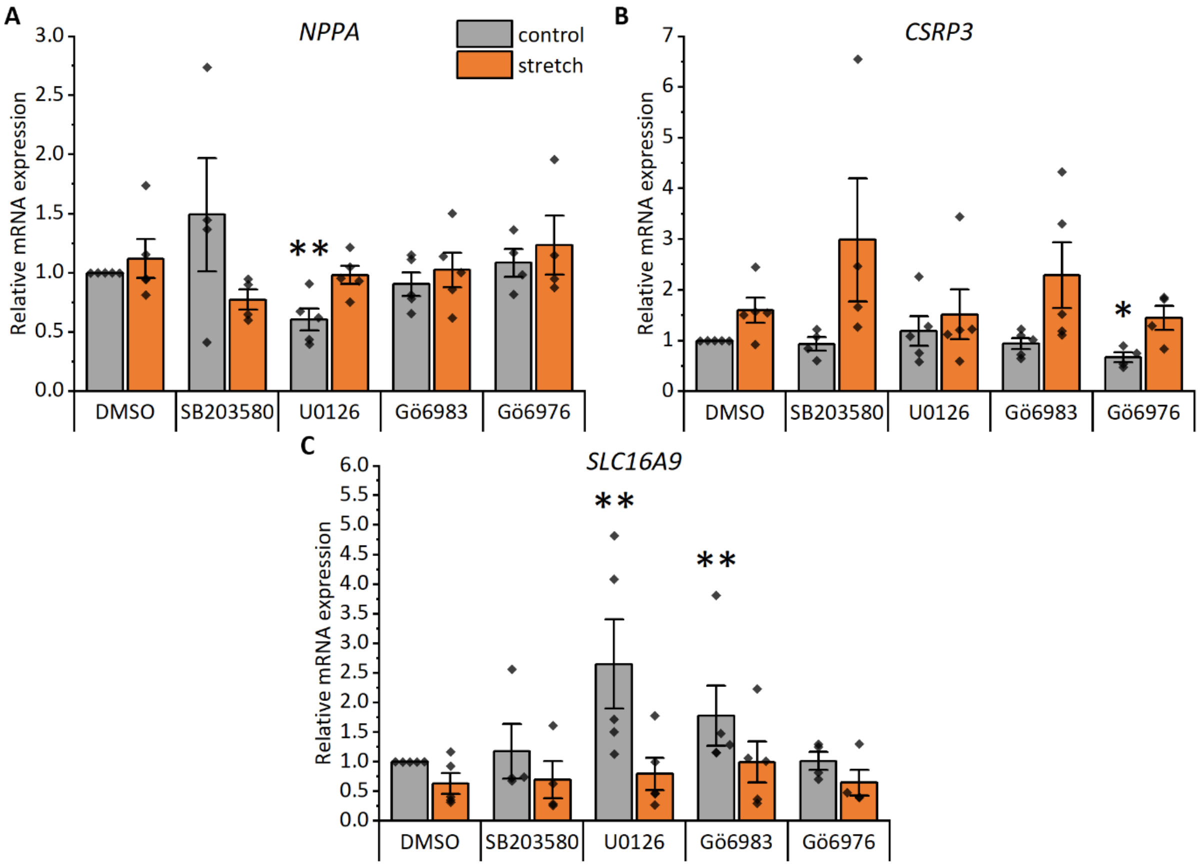

Supplementary Fig. 6. Effects of p38 mitogen-activated protein kinase (p38 MAPK), mitogen-activated protein kinase kinase 1/2 (MEK1/2), and protein kinase C (PKC) inhibitors on NPPA, CSRP3 and SLC16A9 gene expression in hiPSC-CMs. The following inhibitors were utilized: SB203580 at 10 µM to inhibit p38 MAPK, U0126 at 10 µM to inhibit MEK1/2, Gö6983 at 1 µM to inhibit all PKC isoforms, and Gö6976 at 1 µM to inhibit classical PKC isoforms. Natriuretic peptide A (NPPA; A), cysteine and glycine rich protein 3 (CSRP3; B), and solute carrier family 16 member 9 (SLC16A9; C) mRNA expression was measured with qRT–PCR after a 24-h cyclic mechanical stretch. The results are presented as fold change relative to the unstretched control. The data are shown as mean ± standard error of the mean, and values from individual experiments are presented as dots (n=5, except for SB203580 and Gö6976 n=4; where n represents biological replicates of cells from individual differentiations). *$p<0.05$, **$p<0.01$ vs. unstretched DMSO, † $p<0.05$ vs. stretched DMSO, Mann–Whitney U test.

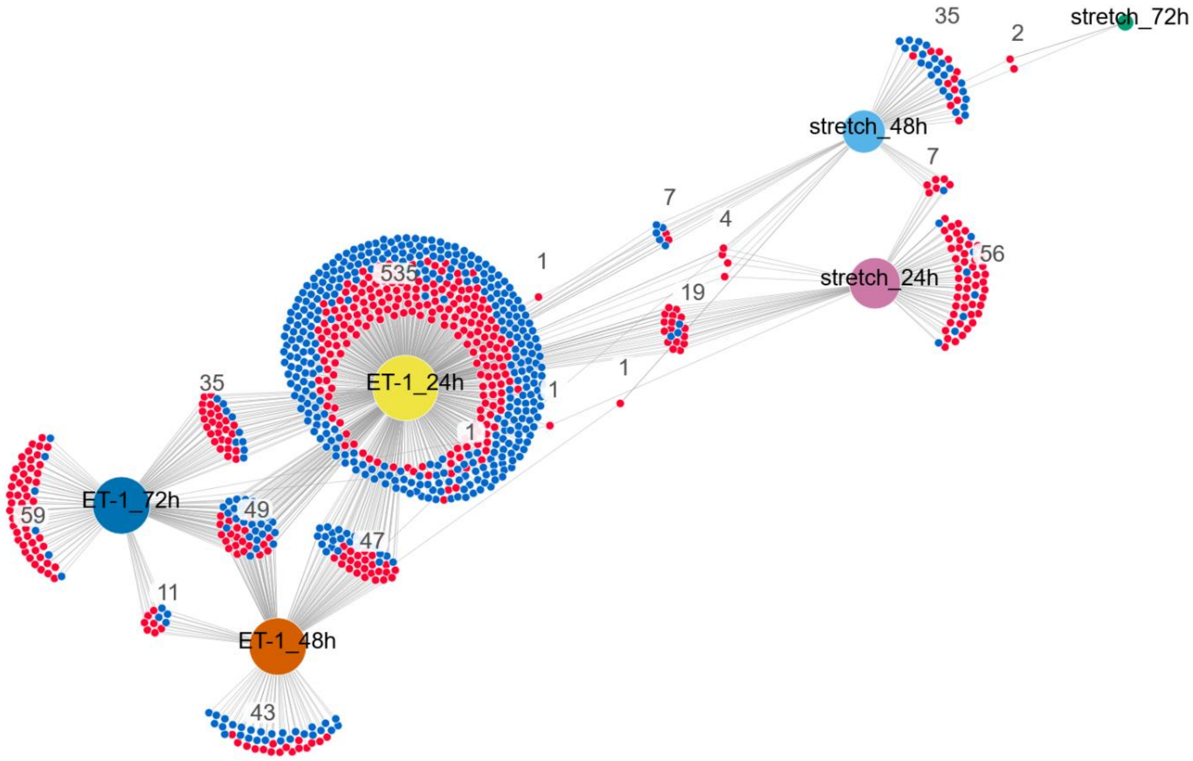

Supplementary Fig. 7. Up- (red nodes) and downregulated (blue nodes) genes in response to endothelin-1 (ET-1) or cyclic mechanical stretch for 24 h, 48 h and 72 h. ET-1 data is from Johansson et al. 2020. Figure was created using DiVenn 2.0 (Sun et al. 2019).

Supplementary Tables

Supplementary Table 1. Used commercial TaqMan® Gene Expression Assays.

**Target**	**Assay number**
18S rRNA, eukaryotic 18S ribosomal RNA	4352930E
ACTA1, actin, alpha skeletal muscle	Hs00559403_m1
ACTC1, actin, alpha cardiac muscle 1	Hs01109515_m1
ACTN1, alpha-actinin-1	Hs00998100_m1
ACTB, beta-actin	4333762T
CSRP3, cysteine and glycine-rich protein 3	Hs00185787_m1
GAL, galanin	Hs00544355_m1
LINC00648, long intergenic non-protein coding RNA 648	Hs06598367_m1
NPPA, natriuretic peptide A	Hs00383230_g1
NPPB, natriuretic peptide B	Hs01057466_g1
PTPRG-AS1, PTPRG antisense RNA 1	Hs04970789_m1
SLC16A9, solute carrier family 16 member 9	Hs00415854_m1
TNNI3, cardiac troponin I3	Hs00165957_m1

Supplementary Table 2. Protein families of the differentially regulated genes of the stretched hiPSC-CMs.

**KEGG identifier**	**Protein family**	**Number of genes**
**UPREGULATED**		**77**
hsa01000	Enzymes	26
hsa04147	Exosome	20
hsa04812	Cytoskeleton proteins	15
hsa03036	Chromosome and associated proteins	8
hsa02000	Transporters	5
hsa04131	Membrane trafficking	5
hsa03019	Messenger RNA biogenesis	5
hsa03029	Mitochondrial biogenesis	4
hsa01009	Protein phosphatases and associated proteins	4
hsa04090	CD molecules	3
hsa03110	Chaperones and folding catalysts	3
hsa03011	Ribosome	3
hsa01002	Peptidases and inhibitors	3
hsa04990	Domain-containing proteins not elsewhere classified	3

hsa03000	Transcription factors	3
hsa00537	Glycosylphosphatidylinositol (GPI)-anchored proteins	2
hsa00199	Cytochrome P450	1
hsa01004	Lipid biosynthesis proteins	1
hsa04050	Cytokine receptors	1
hsa01007	Amino acid related enzymes	1
hsa03016	Transfer RNA biogenesis	1
hsa03041	Spliceosome	1
hsa04040	Ion channels	1
**DOWNREGULATED**		**29**
hsa01000	Enzymes	6
hsa03000	Transcription factors	6
hsa04990	Domain-containing proteins not elsewhere classified	3
hsa02000	Transporters	3
hsa03036	Chromosome and associated proteins	3
hsa03400	DNA repair and recombination proteins	2
hsa04131	Membrane trafficking	2
hsa01009	Protein phosphatases and associated proteins	2
hsa01003	Glycosyltransferases	1
hsa01001	Protein kinases	1
hsa04147	Exosome	1
hsa04040	Ion channels	1
hsa01002	Peptidases and inhibitors	1
hsa04515	Cell adhesion molecules	1

Supplementary Table 3. Fold changes of the common differentially expressed genes of the stretched hiPSC-CMs, NRVMs (data from Rysä et al. 2018) and/or hESC-CMs (data from Ovchinnikova et al. 2018). Only statistically significant values are shown ($p<0.05$ for hiPSC-CMs and NRVMs and $p<0.01$ for hESC-CMs).

external gene name	Fold change, stretch vs control				
	24 h hiPSC-CM	48 h hiPSC-CM	24 h NRVM	48 h NRVM	48 h hESC-CM
TUBB2B	2.7	2.6	2.3	2.0	2.2
CASQ1	1.8	1.6	2.6	1.8	
TIMP1	1.7	1.7	1.5	1.6	
ACAT2	1.8	1.7		1.8	1.8
CSRP3	2.0	1.7		1.5	
TPM2	1.7	1.7		1.5	
CNN1	2.1		4.0	7.4	1.9
MLLT11	1.7		2.7	3.7	1.6
NPPB	4.6		1.5	1.7	2.0
ACTN1	1.6		2.8	5.0	
GADD45G	1.9		1.7	2.7	
GPATCH4	1.6		1.7	1.5	
TAGLN	3.6		2.0	1.7	
TNFRSF12A	2.1		2.6	3.9	
TUBB6	1.8		1.7	2.5	
KRT18	1.9		1.5		
SYPL2	1.5		1.7		
PEA15	1.8			1.8	1.8
CYSTM1	1.6			1.5	
NME1	1.7			1.8	
PRSS23	2.1			1.5	
TUBB2A	2.1			1.7	
MASP1	2.0		0.6	0.5	1.6
CKB	2.2				1.7
TUBA4A	2.0				2.4
BOK	1.5				1.5
PTPRN		4.2	1.8	2.2	
RCAN1		1.5	1.9	2.6	

ENO3		1.6		0.5	1.6
DUSP13		2.8			2.4
CES1		2.0		0.4	
GPCPD1		0.6	0.6	0.5	
ZNF519		0.6	0.6	0.6	0.6
ADAM22		0.6			0.6
EGR1		0.5			0.5
EGR3		0.5			0.6
HELLS		0.6			0.5
HMGB2		0.7			0.6
MKI67		0.6			0.2
SKA3		0.5			0.4
TTYH2		0.6			0.5
DLG2	0.6			0.6	0.5
PLCG2	0.7				0.4
POLQ	0.7				0.5
SDK1	0.6				0.6
RAD51AP1	0.6				0.6
LINC00648	0.5				0.7

Supplementary Table 4. The predicted interaction pairs for the differentially expressed lncRNAs of the stretched hiPSC-CMs.

**LncRNA**	**Interaction Pair**
AZIN1-AS1	PNN, SNRPN, CCDC32, KDM6A, FAM96B, SC5D, EGLN3, RHOH, AP003354.2, AC008124.1
LAMTOR5-AS1	KIF1B, MAPK6, ATP5S, RPL11, SLC1A5, GOSR2, SRD5A3, PPFIA1, U2AF1, ZNF621, PPIAP24, RNU6-179P, RNA5-8S5, RN7SL2, RN7SL703P, AL355488.1, U2AF1L5, AL139099.4, TDGF1, RNA28S5
LINC00648	SCAMP1, ARMC1, PRDM15, RPS27A, TMEM50A, AC002075.2, RPS6KC1, SYNPR-AS1
LINC01341	TKT
PTPRG-AS1	SRSF7, AMPD2, RNA28S5

Supplementary Table 5. Fold changes of the common differentially expressed genes of the stretched hiPSC-CMs and ET-1-treated hiPSC-CMs (data from Johansson et al. 2020 and Aggarwal et al. 2014). Only statistically significant values are shown (p<0.05).

	Current study		Johansson et al. 2020			Aggarwal et al. 2014
**Gene name**	**24 h stretch**	**48 h stretch**	**24 h ET-1**	**48 h ET-1**	**72 h ET-1**	**18 h ET-1**
TUBB2B	2.73	2.62	3.17			
SYNGR3	2.28	2.11	2.41			
ACTC1	1.94	1.61	2.51			
ACAT2	1.84	1.70	2.42			
NPPB	4.64		7.48		2.12	5.64
ACTA1	11.41		12.59			5.54
TAGLN	3.60		4.75			6.37
PLA2G3	2.46		3.29			
KRT8	2.42		2.14			3.67
TUBB2A	2.07		2.41			3.08
MASP1	2.04		3.28			4.19
KRT18	1.93		2.07			4.59
FGFBP3	1.91		2.00			
PEA15	1.81		2.50			
RPL22L1	1.79		2.26			
ENO1	1.76		2.08			
TUBB6	1.75		2.14			
MGST1	1.70		2.25			
NME1	1.68		2.16			
NES	1.57		2.07			
GBE1	1.51		3.20			
PDYN		3.15	6.56	4.57	2.61	4.49
LINC00702		3.77	2.91		2.12	
DUSP13		2.82	2.00			
CREB3L1		2.07	2.30			
GABRP		2.90		2.30		
PLCG2	0.67		0.44			
SLC35E2A	0.61		0.49			
DLG2	0.60		0.34			
MXD3		0.57	0.32			
LINC01341		0.56	0.41			
CFAP70		0.56	0.38			
UNC80		0.43	0.30			
CRIP1		0.40	0.41			
EGR3		0.47				5.63
EGR1		0.52				4.80
GADD45G	1.90					4.40

TNFRSF12A	2.09					4.28
RCAN1		1.52				4.11
TUBA4A	1.99					4.04
PRSS23	2.09					3.18
NOP16	1.53					3.10
ACTN1	1.56					3.10
HELLS		0.63				0.33
ADAM22		0.60				0.32
SLC16A9		0.20				0.32
TTYH2		0.64				0.31
RAD51AP1	0.55					0.26
CDKN2C		0.51				0.19
LINC00648	0.50					0.19

References

Aggarwal P, Turner A, Matter A, Kattman SJ, Stoddard A, Lorier R, Swanson BJ, Arnett DK, Broeckel U. RNA expression profiling of human iPSC-derived cardiomyocytes in a cardiac hypertrophy model. PLoS One. 2014;9(9):e108051. doi: 10.1371/journal.pone.0108051.

Johansson M, Ulfenborg B, Andersson CX, Heydarkhan-Hagvall S, Jeppsson A, Sartipy P, Synnergren J. Cardiac hypertrophy in a dish: a human stem cell based model. Biol Open. 2020;9(9):bio052381. doi: 10.1242/bio.052381.

Ovchinnikova E, Hoes M, Ustyantsev K, Bomer N, de Jong TV, van der Mei H, Berezikov E, van der Meer P. Modeling Human Cardiac Hypertrophy in Stem Cell-Derived Cardiomyocytes. Stem Cell Reports. 2018;10(3):794-807. doi: 10.1016/j.stemcr.2018.01.016.

Rysä J, Tokola H, Ruskoaho H. Mechanical stretch induced transcriptomic profiles in cardiac myocytes. Sci Rep. 2018;8(1):4733. doi: 10.1038/s41598-018-23042-w.

Sun L, Dong S, Ge Y, Fonseca JP, Robinson ZT, Mysore KS, Mehta P. DiVenn: An Interactive and Integrated Web-Based Visualization Tool for Comparing Gene Lists. Front Genet. 2019;10:421. doi: 10.3389/fgene.2019.00421.
